# Vascular injury due to a peripherally inserted central catheter in a neonate born during the 24^th^ week of gestation

**DOI:** 10.1002/ccr3.5991

**Published:** 2022-06-19

**Authors:** Tomonori Kurimoto, Ryo Yonishi, Hirotaka Uehara, Hayato Fukuzato, Satoko Seki, Yoshikazu Shimoji, Okitaka Nakayama, Chiaki Ohba, Iwao Nakazato, Tatsuo Ohshiro

**Affiliations:** ^1^ Neonatology, Okinawa Prefectural Nanbu Medical Center and Children's Medical Center Haebaru Japan; ^2^ Pathology, Okinawa Prefectural Nanbu Medical Center and Children's Medical Center Haebaru Japan

**Keywords:** extremely low birth weight, neonate, peripherally inserted central venous catheter, vascular injury

## Abstract

We investigated a case of a peripherally inserted central venous catheter associated with iliolumbar venous extravasation in an infant. Hyperosmolar infusion and calcium gluconate caused phlebitis and vascular perforation. Daily monitoring of the catheter length at the insertion site and serial radiography may aid in detecting catheter movement.

## Abstract 2

Daily monitoring of peripherally inserted central venous catheter length at the insertion site and serial radiography may aid in detecting catheter movement.

## 1 INTRODUCTION

Infants with extremely low birth weight (ELBW; <1000 g at birth) frequently require intravenous access for an extended period of time for the administration of total parenteral nutrition, hyperosmolar solutions, cardiac inotropic agents, and certain medications or solutions that are chemical irritants, especially when these premature infants temporarily have inadequate gastrointestinal tract intake. Peripherally inserted central venous catheters (PICCs) are widely used and are valuable in the management of infants with ELBW. PICCs may lead to complications associated with their placement and use such as infections, catheter thrombosis, extravasation, catheter malposition, fractured catheters, pleural effusions, cardiac tamponade, and death.^1^ However, lower extremity complications due to central venous catheters have not been well documented.

This report describes the case of an infant with ELBW and a PICC in the inferior vena cava inserted from the lower extremity veins. The infant developed an acute abdomen as a result of intraperitoneal extravasation of parenteral nutrition.

## 2 CASE REPORT

A premature female newborn weighing 708 g was born during the 24^th^ week of gestation and admitted to the pediatric intensive care unit with a diagnosis of respiratory distress syndrome. Thus, the infant needed exogenous surfactant therapy and mechanical ventilatory support. A 27‐gauge double‐lumen polyurethane PICC line (Covidien Japan) was inserted from the right great saphenous vein. Indomethacin was administered three times for patent ductus arteriosus, but the ductus arteriosus could not be closed. Therefore, it was clipped on the sixth day of the infant's life. Enteral feeding did not advance; therefore, the PICC line was continuously administered.

An increased leukocyte count was noted on the 15^th^ day of life (white blood cells [WBCs], 15,300/mm^3^; hemoglobin, 12.3 g/dl; hematocrit, 35.1%; platelets, 541,000/mm^3^; granulocytes, 46.3%; monocytes, 17.5%; lymphocytes, 36.2%; and C‐reactive protein [CRP], 0.01 mg/dl). Catheter‐associated infection was suspected and the PICC was repositioned from the right great saphenous vein to the left great saphenous vein. The infant was given antimicrobial therapy with cefazolin.

The position of the PICC tip on the right side of the fifth lumbar vertebra was confirmed with radiography. From the 13^th^ day onward, steroids were used for the maintenance of blood pressure. On the 17^th^ day, lowering of blood pressure, oliguria, and a rise in serum creatinine level (1.82 mg/dl) were noted. The infant eventually developed anuria.

On the 22^nd^ day, the patient was diagnosed with renal failure because of infection, and peritoneal dialysis was initiated. Catecholamine and fluid therapy were also administered to maintain blood pressure. Blood test results on the same day showed that the CRP level had risen to 7.6 mg/dl (day 22: WBC count, 8000/mm^3^; on day 23: WBC count, 5900/mm^3^ and CRP level, 20.5 mg/dl).

The radiograph revealed that the abdomen was gasless; however, it did not indicate a clear intestinal perforation. Necrotizing enterocolitis was suspected and antibiotics (ceftazidime at 100 mg/kg and vancomycin at 15 mg/kg per day) were administered. Gram‐negative rot was detected on blood culture examination, and the antimicrobial agent was changed from ceftazidime to meropenem, administered at 120 mg/kg per day. PICC replacement was not possible because of marked edema, inability to see the blood vessels, and unstable blood pressure. The infant died of septic shock on the 26^th^ day.

## 3 AUTOPSY

**FIGURE 1:**
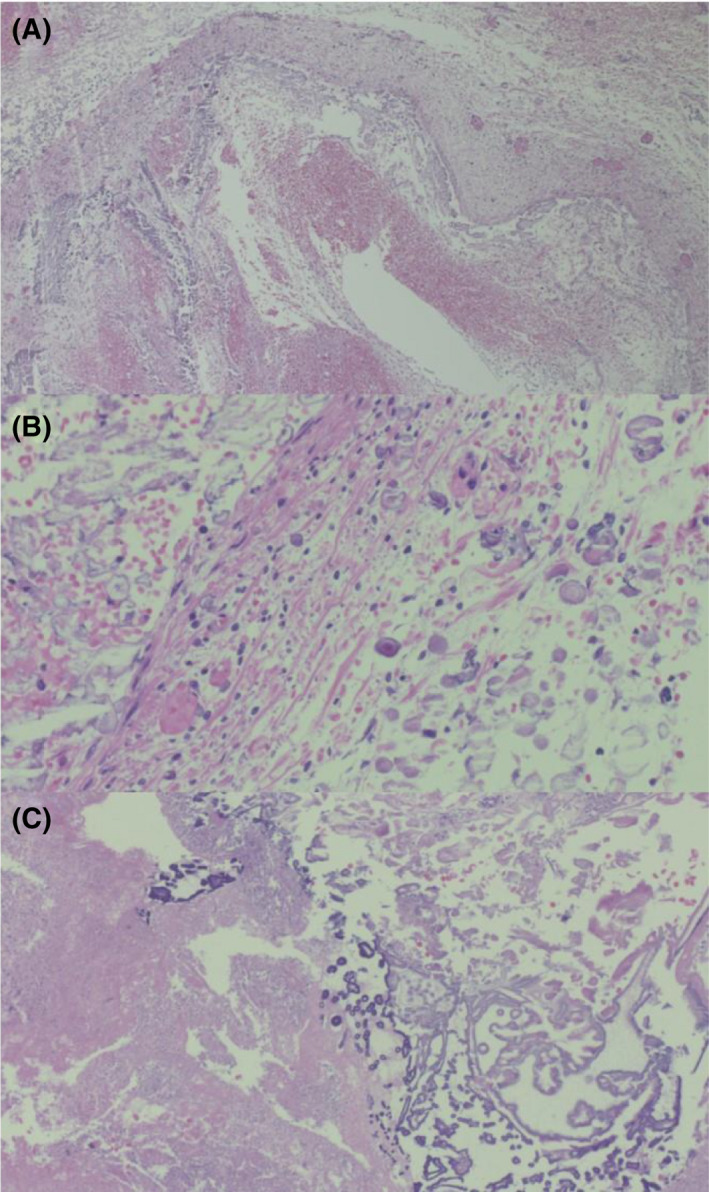
Light microscopy (hematoxylin and eosin). (A) Left iliopsoas vein: necrosis and inflammatory cell infiltration (×200). (B) Left iliopsoas vein: calcification of the venous wall. (×400). (C) Left iliopsoas: calcification in necrotic tissue (×200)

Laparotomy revealed a collection of purulent ascites and an abscess in the left iliopsoas muscle and abdominal wall. The following findings were not noted: perforation of the intestine, clear necrosis or inflammation of the intestinal mucosa, or inflammation in the serosa of the intestine. Necrotic tissue and neutrophilic infiltration were observed around the iliolumbar vein on the abdominal wall‐side of the left iliopsoas muscle (Figure 1A). Calcification was observed around the necrotic area (Figure 1B).

An abscess, which seemed to be a chemical inflammation caused by the drugs (i.e., calcium gluconate and hyperosmolality fluid), had primarily formed in the iliolumbar vein circumference. Calcification deposition was also noted (Figure 1C). The inflammation may have spread from the iliolumbar vein to the intestine and peritoneum, thereby causing bacteremia. No necrosis was observed in the intestine. Inflammation was observed primarily on the serosal side rather than on the intestinal lumen side. The source of infection was not the intestine.

## 4 DISCUSSION

In this study, we describe a case of iliolumbar venous extravasation caused by the movement of a peripherally inserted central venous catheter in an infant. The infant developed an acute abdomen as a result of intraperitoneal extravasation of parenteral nutrition. Hyperosmolar infusion and calcium gluconate caused phlebitis and vascular perforation. The infant died of septic shock on the 26^th^ day. The pathological examination revealed the presence of necrotic tissue, neutrophil infiltration, and calcification around the iliopsoas vein on the abdominal wall of the left iliopsoas muscle. The source of the infection was thought to be an abscess around the iliopsoas vein, which could represent chemical inflammation caused by the drug.

On the 15^th^ day of the infant's life, a PICC was inserted in the left great saphenous vein for suspected sepsis. Reverse blood flow occurred at the time of insertion, and the catheter tip was at the level of (and on the right side of) the fifth lumbar vertebra, which confirmed that the catheter tip reached the inferior vena cava or left common iliac vein.

**FIGURE 2:**
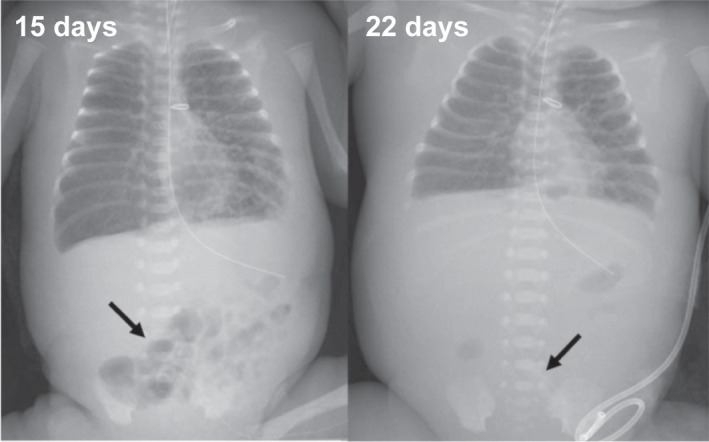
PICC tip position on the radiograph. The arrow points to the tip of the PICC from the left great saphenous vein on the 15^th^ and 22^nd^ day

With reference to the PICC tip position on the 15^th^ day, the X‐P on the 22^nd^ day revealed that the tip had moved to the sixth lumbar vertebra on the left side with a peripheral measurement of approximately 1.5 cm (Figure 2). This migration may have caused the tip of the PICC to enter the iliopsoas vein. Ohki et al.^2^ reported that extension of the lower extremity causes the catheter to move peripherally, followed by flexion of the catheter into the vein that connects to the inferior vena cava. Based on this mechanism, a possibility is that, in the case of our patient, an extension of the lower extremities led to the movement of the catheter tip during spontaneous movement of the child and care given by the nurse.^2^ In addition, an infection caused edema in the subcutaneous tissue (from the insertion site of the PICC to the insertion vessel), and the tip of the PICC may have moved to the peripheral side. Central venous hyperalimentation was subsequently administered through the main route of the PICC, and the osmolality was 816–986 mOsm/L. The osmolality of the fluid administered was greater than 900 mOsm/L. Calcium gluconate (4.25%; 140 mOsm/L) was also administered through the PICC subroutine. Therefore, phlebitis was a possibility.^3^, ^4^

These findings indicated that edema of the cytoplasm, which was caused by increased vascular permeability because of infection, extension, and flexion of the lower extremities, caused the tip of the PICC to move to the peripheral side and to be placed in the iliopsoas vein with a narrow vessel diameter. Hyperosmolar infusion and calcium gluconate administration caused phlebitis and vascular perforation. In addition, fluid may have leaked into the peritoneal cavity, thereby causing inflammation and abscess formation.

If catheter‐related complications are suspected, ultrasonography and lateral radiography may help in diagnosing catheter malposition, which is sometimes missed in frontal radiographs. Daily monitoring of the catheter length at the insertion site and serial radiographs may be helpful in detecting catheter movement before it leads to further complications.

## 5 CONCLUSION

The edema from bacteremia and movement of the lower extremities caused the tip of the PICC to move to the peripheral side and be placed in a narrow vessel. Hyperosmolar infusion and calcium gluconate led to phlebitis and vascular perforation, respectively. This factor eventually led to inflammation and abscess formation in the peritoneal cavity. Daily monitoring of the catheter length at the insertion site and serial radiographs may help detect catheter movement.

## AUTHOR CONTRIBUTIONS

RY, HU, HF, SS, YS, ON, and CO conceptualized this study. TK conceptualized and designed the study, and wrote and edited the manuscript. IN supervised pathological results of the study. TO supervised the study. All authors approve of the final manuscript before submission and have agreed to be accountable for all aspects of this work.

## CONFLICT OF INTEREST

The authors have no conflicts of interest to declare.

## ETHICAL APPROVAL

This case study was conducted in accordance with the provisions of the Declaration of Helsinki, as revised in Tokyo in 2004, and was approved by the Ethics Committee of Okinawa Prefectural Nanbu Medical Center and Children's Medical Center (Haebaru, Japan): approval number R3‐140.

## CONSENT

Written informed consent was obtained from the patient's parents to publish this report in accordance with the journal's patient consent policy.

## Data Availability

Data sharing is not applicable to this article as no datasets were generated or analyzed during the current study.
